# Migration of dual-earner couples: a subjective wellbeing approach

**DOI:** 10.1007/s11150-021-09598-z

**Published:** 2022-01-18

**Authors:** Diana Tam, Arthur Grimes

**Affiliations:** 1 Victoria University of Wellington, Wellington, New Zealand; 2 Motu Economic & Public Policy Research, Wellington, New Zealand

**Keywords:** I31, J12, J16, J61, Migration, Dual-earner couples, Subjective wellbeing, Bargaining

## Abstract

We model push factors that determine the domestic migration decisions for couples, with emphasis on dual-earner different-sex couples. Unlike many prior studies that concentrate on labour market determinants of migration, we place the subjective well-being (SWB) reported by each partner at centre stage. We test whether migration determinants differ depending on whether the female is the main breadwinner in a dual-earner couple. We also test if determinants differ when either the female or the male is the sole earner within a couple. The evidence shows that a couple is more likely to migrate if she reports low SWB in the year prior to migration, with the strength of this effect varying depending on the earnings status of each partner prior to migration. Male SWB does not have the same impact on the migration choice although we find some evidence that pre-migration male wages impact the migration decision.

## Introduction

In an age of geographic mobility,^1^ it is relatively easy to move domestically to other places in search of new job opportunities and better lifestyles. The decision to migrate is more complicated for those in a relationship versus those who are not. A couple must balance the motivations and desires of each partner. For dual-earner couples, in particular, there is a need to consider the career opportunities and outcomes for each partner. We analyse which push factors predict a dual-earner couple’s decision to jointly migrate, as opposed to making a decision to: (a) both stay, or (b) have one partner leave and one stay. In analysing dual-earner couples, we differentiate between couples where ‘she earns more’ and ‘he earns more’; we also compare the migration determinants of dual-earner couples with other couple types (all couples, male sole-earner, female sole-earner). Unlike most prior papers (e.g. Borjas et al., 1992) we highlight the role of subjective wellbeing (SWB) for each partner in the decision.

In a new approach that takes account of psychological set-point theory (Cummins, 2013), we focus on how each partner’s SWB relative to their observed maximal SWB level affects their migration decision. We interact this variable with an indicator of who in the couple earns more to test the hypothesis that a partner with greater bargaining power (in terms of wage contribution) will have greater weight when the couple makes the joint migration decision.

The modelling focuses on push factors in the migration decision (i.e. factors relating to the couple’s existing location). This reflects the presence of discrete moving costs when shifting residence and region, potentially reflecting loss aversion (Kahneman, 2011) meaning that push factors need to be sufficiently large to induce a decision to leave a location. Once the barrier to leave the existing location is overcome, pull factors then determine the choice of new location. We do not analyse the latter choice.

The roles that SWB, plus labour market and other factors, play in the migration decision are tested using internal migration patterns within Australia. Modelling migration within a country avoids the problems caused by legal barriers to migration, although migration costs (including the psychic costs of moving) still affect relocation choices. To check robustness, our estimates employ both discrete choice models (probit and logit) and a linear probability model, with the dependent variable in each case being the probability that a couple jointly migrates.

Our data source is the Household, Income and Labour Dynamics in Australia (HILDA) panel survey. HILDA provides data on earnings, labour force status, individual and household characteristics and on SWB, in the form of self-reported ‘life satisfaction’. We use the term ‘SWB’ interchangeably with life satisfaction in this paper.

We find strong evidence that the female partner’s SWB (but not the male’s) plays an important role in determining the couple’s migration decision. We also observe that this impact may be magnified when she earns more or where she is the sole earner in the couple, though these effects are not statistically significant. The importance of the female partner’s SWB is apparent even when the male is the sole earner within the couple. These results, which are novel with respect to the couple migration decision, underscore the importance of considering the impact of SWB (as well as other factors) on couples’ decision-making.

Section 2 of the paper provides a brief background on a key labour market trend which motivates our analysis and reviews relevant theoretical and empirical literature. Section 3 describes the data. Our empirical methodology and results on the determinants of the couple migration decision are set out in section 4, with discussion and conclusions in section 5.

## Background

### Labour market changes

The past fifty years have seen an increase in women’s labour force participation rates, particularly for married women (Ortiz-Ospina et al., 2018). Associated with this is an increase in dual-earner couples (Costa & Kahn, 2000), while the traditional male ‘head of house’ structure (where the male is the breadwinner and the female specialises in household production) has become less common. In 2009, the traditional structure represented only about a third of families with dependent children in most developed countries (Apps & Rees, 2009). In Australia, the majority of working age couples are dual-earner couples, and this number has been increasing over time. In 2001, 56% of working age couples were dual-income couples, increasing to 66% of working age couples by 2017 (Wilkins et al., 2019).

The change in labour force structure, and of women’s earnings and mobility, is associated with changes in gender roles and social norms. This has ramifications for household decision-making, meaning that at least some women will have greater input into financial and career decisions than would otherwise have been the case (Thomas, 1990; Winkler, 1998). These changes will be reflected as an increase in the woman’s outside option, i.e. the utility she would receive if she left the relationship.

Dual-earner couples must make decisions which influence both partners’ career outcomes. The more crucial decisions include those related to occupation, fertility and migration. Dual-earner couples tend to be less mobile than single-earner couples (Mincer, 1978). Research also suggests that there are gender differences in the career consequences of family migration (Cooke, 2008; Pailhé & Solaz, 2008).

### Subjective wellbeing

A fundamental economic problem is the utility maximisation problem. Utility comprises both pecuniary and non-pecuniary factors. Migration is influenced by both factors: the pecuniary factors include income and employment opportunities, while the non-pecuniary factors may include influences as diverse as the climate, SWB and even human rights (Grimes & Wesselbaum, 2019). Studies of migration choices have historically tended to focus on pecuniary measures as these have traditionally been easier to measure.

More recently, economic studies have also focused on surveyed evaluative SWB (e.g. life satisfaction) as a measure of an individual’s utility.^2^ Many government agencies and researchers now collect information on SWB, including via national panel surveys. The HILDA survey is an Australian example, using a life satisfaction measure of SWB. Multiple studies show that SWB data can be reliably measured and analysed statistically, using standard methods (Dolan et al., 2008; Graham et al., 2018). One extant issue in this literature is whether SWB measures, which are collected using a Likert scale, can be modelled as if they are cardinal (Ferrer-i-Carbonell & Frijters, 2004; Bond & Lang, 2019). We treat this debate as one which can be addressed empirically, especially when SWB is used as an explanatory variable rather than as a dependent variable, and we use both cardinal and ordinal measures in our analysis.

A small number of studies (surveyed in Grimes & Wesselbaum, 2020) consider how SWB affects, and is affected by, migration.^3^ Nowok et al. (2013) find that regional migration tends to be preceded by a significant fall in SWB for both single men and women. Thus they view migration as a ‘path out of unhappiness’. Preston and Grimes (2019) observe that SWB decreases in the year prior to regional migration within Australia for men and women, both for those who are single and for those who are in relationships. They also report that partnered women enjoy a sustained increase in SWB post-migration, while there is no significant increase for their male counterparts. For international migration, Grimes and Wesselbaum (2019) provide evidence that unhappiness at the origin country is a ‘push’ factor for migration, while happiness at the destination country is a ‘pull factor’. None of these studies, however, models the couple’s migration decision which is our focus in this paper.

We construct a measure of SWB that can be used to test how life satisfaction influences the decision to migrate. Recognising that people’s SWB tends towards a mean reverting pattern, Cummins (2013) proposed that individuals have a ‘set-point’ for SWB which he proxied by the average of an individual’s past levels of SWB. Our approach is similar to that taken by Cummins. However, rather than taking the average level of past SWB, we focus on the individual’s maximum recorded level of SWB across the full panel as an aspiration point for each individual against which they evaluate their current SWB position. We construct a variable for an individual’s life dissatisfaction by subtracting their SWB in the previous year from the individual’s maximum recorded level of SWB. The rationale for this approach is that an individual who is relatively dissatisfied (compared with their aspirational self) will be more motivated to migrate and find somewhere better to live with a higher level of SWB.

### Theoretical models of the household

Decision-making is more complicated for couples than for singles given the additional constraints, and this applies when it comes to making household migration decisions (Mincer, 1978; Clark & Davies Withers, 1999; Jacobsen & Levin, 2000). Since couples need to consider the costs and benefits of migration for both partners, couple households are generally less likely to move than singles (Jacobsen & Levin, 2000).

Traditionally, economists modelled family decision-making using a unitary household model (Samuelson, 1956; Becker, 1981). The income pooling concept inherent in unitary models implies anonymity so that the effect of a change in income does not depend on the recipient of that income. Empirical studies, however, have rejected the anonymity assumption in some contexts (Lundberg et al., 1997; Apps & Rees, 2007). Early migration models (e.g. Sjaastad, 1962) were based on a unitary approach with a rational migrant weighing up the present discounted value of expected returns in various possible locations. Polacheck and Horvath (1977) extended the ‘rational migrant’ model to consider the joint nature of the family migration decision. By positing that a household acts to maximise the present value of the lifetime sum of the couple’s earnings, they effectively assumed that partners are indifferent as to who earns, and in what proportion. The implication of their model is that, in some families, there will be ‘tied movers’ or ‘tied stayers’ (Mincer, 1978).

Historically, the couple migration decision has been modelled as being determined by labour market choices of the male partner (Mincer, 1978). One possible reason is that women have historically had lower earnings potential than men. (Other contributing factors may include gender discrimination, social norms and lower social mobility for women.) Earnings-based models have commonly assumed that couples obtain higher household utility if they move for the benefit of the male partner’s career (Cooke, 2008) but the relevance of this approach today in many countries is questionable (Cotter et al., 2011). For instance, Foged (2016) analyses family migration in Denmark, focusing on young Danish-born dual-earner couples, finding that these couples are more likely to migrate if an increase in household earnings potential is disproportionately due to one partner regardless of the partner’s sex. Similarly, Cooke (2013) considers migration in the United States in 1997 and 2007 finding that tied migration is relatively rare, and that tied staying is much more common and experienced equally by men and women.

By contrast with unitary models, non-unitary models explicitly account for unequal power and resource distribution within households (e.g. McElroy, 1990; Carter & Katz, 1997; Lundberg & Pollak, 2008). In these models, each individual maximises a private utility function which depends on both their own outcome and other household members’ outcomes. A key feature in these models is the ‘sharing rule’, which governs the intra-household distribution of household resources (Chiappori, 1988; Chiappori, 1992).

Lundberg and Pollack’s (1993; 2008) ‘separate spheres’ model recognises that the outside option of each partner may be one that is internal to the marriage, defined with respect to separate gender roles. Lundberg and Pollack (2003) applies this ‘separate spheres’ model when considering the location decisions of dual-earner couples. Since each partner maximises their private utility, a couple will jointly migrate if the relocation makes *each* partner better off. A partner may *also* rationally choose to move, even if they expect to be worse off in the new location if that option is better than staying in the present location without their partner. The bargaining approach normally assumes that, once a household decision has been arrived at, that decision is binding and can be costlessly enforced (Lundberg & Pollak, 2003; Abraham et al., 2010). In the migration context, the assumption that the decision will be adhered to, once implemented, is reasonable to adopt given the costs of moving again.

While bargaining models have been used to analyse couples’ location decisions (Jacobsen & Levin, 2000; Lundberg & Pollak, 2003; Pailhé & Solaz, 2008; Abraham et al., 2010; Rabe, 2011; Foged, 2016), empirical applications to couple migration remain few. Empirical testing is challenging because it is not possible to accurately measure either bargaining power or the outside option. Studies have relied upon proxies for bargaining power such as relative incomes, marriage ages, whether the mother or father is entitled to receive child benefits, divorce laws, and a range of factors that affect the attractiveness of a partner’s outside option (Lundberg et al., 1997; Ward-Batts, 2001; Chiappori et al., 2002). Measurement of the outside option is difficult since all factors that affect utility are relevant in determining that option.

Our approach reflects a Nash bargaining model (e.g. Manser & Brown, 1980; McElroy & Horney, 1981) in which the outside option of each partner is the utility the partner will receive in the event they are unable to reach agreement to migrate jointly. This utility is proxied (inversely) by their existing level of life dissatisfaction in the current location. The distribution of bargaining power is likely to depend on factors such as the job opportunities that a partner may obtain in the labour market, the chances of entering a new relationship, and the legal frameworks relating to divorce, divorce settlements and eligibility for social welfare payments. Some of these factors (e.g. legal frameworks) are exogenous to the couple while others are unobservable (e.g. chances of a new relationship). Our proxy for bargaining power is the labour market position of each partner reflected in their relative earning power (as in Bozon, 1991). We hypothesise that migration propensity will be enhanced when either or both partners is more dissatisfied with life in the current location so they have more to gain from migration. Additionally, reflecting bargaining power, we test whether the partner who earns more within the couple is able to increase the weight placed on their level of life dissatisfaction in determining the couple’s migration choice.

## Data and descriptive statistics

We use the same dataset as Preston and Grimes (2019) who used the Household Income and Labour Dynamics in Australia (HILDA) survey to study migration outcomes (rather than the determinants of migration). As in that study, we define migration as a residential move of 25 km or more within Australia. Moves to other countries are excluded from the dataset. The data come from 14 waves of the HILDA survey, from 2001 to 2014; wave 1 is dropped from the analysis given that couples are defined as at the prior wave and also given the inclusion of lagged terms in the specification. (We do not include further lags in our modelling in order to maximise the length of the panel.)

HILDA tracks a representative sample of Australians over the course of their lifetimes. Individuals are interviewed on an annual basis, and asked about their household and labour market experiences over the previous year. The data collected includes address changes (used to define migration) plus demographic and socioeconomic indicators such as age, gender, ethnicity, children, earnings, education and employment status. The survey’s SWB measure records an individual’s answer to the question: “*All things considered, how satisfied are you with your life?”* An individual may respond with a whole number from 0 to 10, with 0 being “*Totally dissatisfied*” and 10 being “*Totally satisfied*.” HILDA allows for the construction of data for both members of a couple in each wave, enabling us to include partner variables in each year.

The dataset comprises individuals who are aged between 25 and 59 years old when observed in the year of (potential) migration. The reason for the age restriction is to exclude migration that follows either the completion of studies or around retirement. Our primary focus is dual-earner couples, defined as different-sex^4^ couples (married or cohabiting) where each partner is in the workforce. To create ‘couple’ observations, we take each observation of females in relationships, and combine them with data on the characteristics of their male partner. For both partners, we obtain information on age, gender, weekly wages (CPI-adjusted), and education level. For robustness checks, we also obtain information on whether either partner was made redundant or fired over the preceding year. Characteristics at the couple level include: the number of dependent children in the household (split into two age groups), marital status, and whether the couple owns a home. We expect that migration will be reduced if a couple owns a home (Mok, 2007) or if they have more dependent children, reflecting increased costs of moving. Couples who are legally married are likely to attach a higher cost to ‘splitting up’, and are therefore more likely to make a joint migration decision. However, they may also have higher costs of moving so the effect of marriage on migration propensity is indeterminate.

Table 1 provides summary statistics for three different categories of people in the dataset: all individuals, those in couple relationships (married or cohabiting), and those in dual-earner couple relationships only. The total dataset comprises 59,927 individual-year observations including all singles, couples, and those in relationships which do not fit our strict definition of ‘couple’. There are 15,084 total couple-year observations and 10,693 couple-year observations for dual-earner couples covering 1,419 couples.

**Table 1:** Descriptive statistics: individuals and couples

	All		Couples		Dual-earner couples	
Variable	Men	Women	Men	Women	Men	Women
Age	43.3 (9.65)	43.2 (9.59)	45.7 (8.16)	43.5 (8.00)	45.7 (7.79)	43.6 (7.70)
Relationship (=1 if in a relationship)	0.59 (0.49)	0.59 (0.49)	[1.00]	[1.00]	[1.00]	[1.00]
Highest education						
Year 11 and below	0.22	0.31	0.21	0.31	0.18	0.26
Year 12	0.12	0.15	0.09	0.15	0.09	0.14
Cert III or IV	0.31	0.15	0.32	0.14	0.32	0.15
Advanced diploma, diploma	0.10	0.10	0.11	0.11	0.11	0.11
Bachelor, Honours	0.15	0.16	0.15	0.16	0.16	0.19
Grad diploma, certificate	0.06	0.09	0.07	0.10	0.08	0.11
Postgrad	0.05	0.04	0.06	0.03	0.06	0.04
Employed (=1 if employed)	0.88 (0.32)	0.73 (0.44)	0.91 (0.28)	0.75 (0.43)	[1.00]	[1.00]
Hours/week (hours per week worked in all jobs)	44.6 (12.4)	32.1 (14.1)	45.7 (12.3)	30.2 (14.1)	45.9 (12.2)	30.1 (14.0)
Part-time work (less than 40 h per week)	0.27 (0.44)	0.67 (0.47)	0.23 (0.42)	0.72 (0.444	0.23 (0.42)	0.73 (0.44)
Wages (CPI-adjusted weekly wage in AUD)	1104 (982)	610 (626)	1191 (1030)	580 (601)	1268 (960)	771 (577)
Ever migrated	0.20 (0.40)	0.20 (0.40)	0.16 (0.36)	0.16 (0.36)	0.15 (0.36)	0.15 (0.36)
Observations	27,841	32,086	15,084	15,084	10,693	10,693

Standard deviations are shown in brackets. Wages of non-earners (in the All and Couples columns) are set to zero

Notable features are as follows. Those in relationships tend to be older than their single counterparts, particularly so for men. Women are more likely to have received a Bachelor’s degree or graduate diploma compared with their partners but the opposite holds for post-graduate degrees. Men are more likely to be employed than women, with women more likely to be employed part-time. Across all categories, men on average work more hours per week than women. Average weekly wages are higher for men and this occurs even for dual-earner couples where, by definition, both partners are working. (The average weekly wage figure for non-workers in the All and Couples columns of Table 1 is set to zero for those not employed.) Individuals in couples are less likely to have ever migrated compared with singles, while individuals in dual-earner couples are even less likely to migrate than those in other couples, potentially reflecting the difficulties in coordinating two careers while migrating.

Table 2 provides summary statistics on mean SWB for the same three categories (all individuals, all couples, and dual-earner couples only). For comparison, we also present mean SWB for single-earner couples, delineating between couples where the male partner works, and those where the female partner works. For dual-earner couples, we present data for sub-groups based on who earns more within the couple. The dummy variable for ‘she earns more’ equals one if her weekly wages exceed his weekly wages; this occurs for 25.9% of dual-earner couples.

**Table 2:** Mean life satisfaction by sex, earnings, education, and age differentials

Sample	Mean life satisfaction (s.d.)	Observations
*All individuals*		
All individuals		
Men	7.70 (1.06)	27,841
Women	7.81 (1.10)	32,086
*All couples (and single-earner subsets)*		
All couples		
Men	7.84 (0.98)	15,084
Women	7.98 (0.96)	15,084
Single-earner couples—he earns, she doesn’t		
Men	7.89 (0.93)	3155
Women	8.09 (1.01)	3155
Single-earner couples—she earns, he doesn’t		
Men	7.59 (1.21)	1552
Women	7.90 (0.93)	1552
*Dual-earner couples (and sub-sets)*		
Dual-earner couples		
Men	7.89 (0.90)	10,693
Women	7.97 (0.89)	10,693
Dual-earner couples—he earns more		
Men	7.90 (0.89)	7097
Women	7.98 (0.89)	7097
Dual-earner couples—she earns more		
Men	7.83 (0.93)	2772
Women	7.93 (0.88)	2772

Mean life satisfaction for Table 2 is calculated by taking the mean of reported overall life satisfaction for an individual across all waves in which the individual is observed

Consistent with existing SWB literature (Dolan et al., 2008), women tend to report greater levels of life satisfaction than men, and those in relationships tend to report greater life satisfaction than those who are not. For single-earner couples, both men and women tend to have higher SWB when he works but she does not (compared with the scenario where she works, but he does not). Men in single-earner couples on average have lower SWB when she works, but he does not. A similar result holds across dual-earner couples in relation to who earns more, although the differences are not as stark. These differences are, of course, purely associative and so should not be interpreted causally.

Given that migration may be one path ‘out of unhappiness’ (Nowok et al., 2013), we form a variable for life dissatisfaction in the current location, $LD_{i,t - 1}$. This variable contains both an individual-specific fixed component of SWB and a changing component. The fixed component of $LD_{i,t - 1}$ is the maximum level of SWB ($LS_i^{Max}$) observed for the individual over the full course of the panel. We then subtract the individual’s level of SWB in the year prior to migration from $LS_i^{Max}$. Thus $LD_{i,t - 1}$ is a measure of a person’s prior period *life dissatisfaction*; it will be positive if the person is not at their maximal level of observed life satisfaction and will increase in value the more dissatisfied that she is.

Table 3 presents the proportions of females and males within three couple categories (all couples, dual-earner couples, and dual-earner couples where she earns more) according to their levels of life dissatisfaction. (We omit those for whom $LD_{i,t - 1}$ > 5 who comprise less than 0.3% of the sample.) In each case, between 30% and 34% of partners are at their peak life satisfaction ($LD_{i,t - 1}$ = 0) and we observe no material differences in proportions whether delineated by sex or by couple type.

**Table 3:** Distributions of life dissatisfaction by couple type (%)

	0	1	2	3	4	5
Percentage of females within						
All couples	31.7	41.2	18.6	5.2	1.8	1.0
Dual-earner couples	30.5	43.7	18.7	4.7	1.5	0.7
Dual-earner couples and she earns more	30.2	45.0	18.8	4.4	1.1	0.4
Percentage of males within						
All couples	33.1	41.5	17.7	4.8	1.7	0.7
Dual-earner couples	33.4	43.4	17.2	4.0	1.3	0.5
Dual-earner couples and she earns more	31.5	43.1	18.4	4.4	1.7	0.7

N for couples = 15,084; N for dual-earner couples = 10,693; N for dual-earner couples and she earns more = 2772. Percentages for life dissatisfaction >5 omitted due to small N

The propensity to migrate in period t, conditional on $LD_{i,t - 1}$, is shown in Table 4 for the same categories. Two features are noticeable. The first feature is that people who are most satisfied with their lives in period t-1 ($LD_{i,t - 1}$ = 0) have a higher propensity to migrate than moderately unhappy people (1 ≤ $LD_{i,t - 1}$ ≤ 3). The reduced propensity to migrate of moderately unhappy people reflects the psychological literature that people suffering from symptoms of depression or stress can display considerable inertia when making decisions (APA, 1994). The second feature is that once dissatisfaction becomes very large ($LD_{i,t - 1}$ ≥ 4) the migration propensity jumps sharply, consistent with a very strong push factor that contributes to migration and which outweighs the effect of inertia. An implication of the patterns that we observe in Table 4 is the need to control for a peak happiness effect (i.e. $LD_{i,t - 1}$ = 0 relative to $LD_{i,t - 1}$ > 0) in our estimates.

**Table 4:** Joint migration rate by couple type and life dissatisfaction prior to migration (%)

	0	1	2	3	4	5
Females						
All couples	3.4	2.1	2.4	2.9	4.9	3.5
Dual-earner couples	3.3	2.1	2.0	2.6	5.7	6.3
Dual-earner couples and she earns more	2.3	2.6	1.5	1.7	9.7	8.3
Males						
All couples	3.8	2.0	2.2	2.5	3.6	0.9
Dual-earner couples	3.6	2.0	2.3	1.2	5.1	1.9
Dual-earner couples and she earns more	3.5	2.1	1.2	0.8	6.5	5.3

N for couples = 15,084; N for dual-earner couples = 10,693; N for dual-earner couples and she earns more = 2772. Percentages for life dissatisfaction >5 omitted due to small N

## Methodology and results

Our core focus is to estimate the determinants of joint migration for dual-earner couples (noting the rarity of single partner migration in our sample). We also estimate joint migration propensity for other couple types so that we can observe whether migration determinants differ for couples with different characteristics. We hypothesise that, conditional on a range of personal and couple characteristics, migration depends on three focal variables: (his and her) wages, (his and her) level of life dissatisfaction, and a binary variable for each partner representing if they are dissatisfied (i.e. if $LD_{i,t - 1}$ ≥ 0). As noted above, the third of these variables reflects inertia in decision-making of people who are dissatisfied with their life. In addition, reflecting the possibility that the higher income earner has greater bargaining power, we hypothesise that the effect on migration of being dissatisfied differs depending on whether she earns more. Thus our core equation is of the form:

1 $$\Pr \left( {M_{it} = 1} \right) = {{{\mathrm{f}}}}\left( {\begin{array}{ll} {\alpha + \beta _1\left( {x_{f,t - 1}} \right) + \beta _2\left( {x_{m,t - 1}} \right) + \beta _3\left( {x_{h,t - 1}} \right)} \\ \,\,\,\, { +\, \lambda _w + \lambda _r + \beta _4\left( {LD_{f,t - 1}} \right) + \beta _5\left( {LD_{m,t - 1}} \right)} \\\,\,\,\, { +\, \beta _6\left( {Dissatisfied_{f,t - 1}} \right) \ast \left( {\Delta WAGE_{t - 1}} \right)} \\\,\,\,\, { +\, \beta _7\left( {Dissatisfied_{m,t - 1}} \right) \ast \left( {\Delta WAGE_{t - 1}} \right)} \end{array}} \right) + {\it{\epsilon }}_{it}$$

$M_{it}$ is an indicator for whether or not individual $i$ jointly migrates with their partner in year *t* (with the subscript *i* decomposed into *f* and *m*, representing the female and male partners respectively); *x*_*f*_ and *x*_*m*_ are vectors of personal characteristics comprising weekly wages, age and two levels of education (high for people with a tertiary qualification, and low for those with a secondary school qualification or below, with low being the omitted category); *x*_*h*_ is a vector of (shared) couple characteristics comprising home ownership, number of dependent children split between preschool children (0 to 4 years) and older dependents (5 to 24 years so including those of tertiary education age).

Each of the life dissatisfaction variables *(*$LD_{f,t - 1}$ and $LD_{m,t - 1}$) is included by itself and in binary form. The binary variable is denoted $Dissatisfied_{i,t - 1}$, where $Dissatisfied_{i,t - 1} = 0$ if $LD_{i,t - 1} = 0$ and =1 otherwise. This binary variable is interacted with a “she earns more” dummy, denoted in (1) by Δ*WAGE*, where Δ*WAGE* = 1 if she has higher weekly wages than he does (=0 otherwise). The “she earns more” dummy is also included separately in the equation. All characteristics are measured as at *t*−1, the year prior to potential migration. ${\it{\epsilon }}_{it}$ is a composite error term decomposed into *c*_*i*_ (a term capturing unobserved individual heterogeneity) and $u_{it}\sim IN\left( {0,\sigma _u^2} \right)$, a random error term.

In all our estimates, we include indicators for each wave to control for year fixed effects (*λ*_*w*_), and indicators for regions to control for region fixed effects (*λ*_*r*_). Given our concentration on push factors for migration, the region fixed effects are particularly important. If a couple is initially situated in a low wage or low SWB region, they will potentially have greater gains from migration than will couples in more favourable areas. Thus the region fixed effects proxy, *inter alia*, for potential pull effects from other regions.

The dependent variable in each of our regressions is a binary variable for joint migration of the couple. Given this limited dependent variable, we check robustness of our estimates to three estimation techniques: probit, logit and generalised least squares (i.e. a linear probability model), each with random effects. (Fixed effect models omit all couples who do not migrate over the sample period so are not suitable for the analysis. We note, however, that the $LS_i^{Max}$ component of the $LD_{i,t - 1}$ variable represents a fixed effect for the life satisfaction of each partner.) Standard errors are clustered on couples.

Table 5 presents estimation results for dual-earner couples based on Eq. (1) using each of the three estimation techniques. Variables significant at the 5% (and 1%) level are indicated by asterisks. Prior to discussing the focal (SWB and wage) variables, we note that the estimated relationships for the control variables are consistent with standard expectations regarding the migration decision. Couples are less likely to migrate if they own a house. They are also less likely to migrate if they have school-aged children while the presence of pre-school children does not significantly alter the migration probability. A couple is less likely to migrate if the woman is older, but there is no significant effect in relation to the man’s age. However, the ages of the two partners are highly correlated (r = 0.89), so the female age is an indicator of an overall age effect. These results are consistent across all three estimation methods. Couples who are legally married are less likely to migrate than other couples (but only significant in the linear probability model). A high level of education (of each partner) raises the probability of migration (significant only in the probit and logit models). The female education result modifies findings in previous studies that the female partner’s education has an insignificant impact on household migration once the male partner’s education has been controlled for (e.g. Lichter, 1982; Nivalainen, 2004; Swain & Garasky, 2007; Compton & Pollak, 2007).

**Table 5:** Probability of joint migration for dual earner couples

	Probit	Logit	GLS (Linear probability)
Control variables			
Her age	−0.0215**	−0.0489**	−0.0021**
	(0.0076)	(0.0172)	(0.0008)
His age	−0.0043	−0.0082	−0.0012
	(0.0071)	(0.0159)	(0.0008)
Her education	0.1253*	0.2911*	0.0054
	(0.0593)	(0.1355)	(0.0050)
His education	0.1619*	0.3600*	0.0078
	(0.0673)	(0.1548)	(0.0056)
Dependent children aged 0 to 4	0.0106	0.0235	−0.0036
	(0.0476)	(0.1024)	(0.0043)
Dependent children aged 5 to 24	−0.0977**	−0.2113**	−0.0059**
	(0.0192)	(0.0456)	(0.0013)
Married	−0.1005	−0.1301	−0.0428*
	(0.1157)	(0.2488)	(0.0187)
Homeownership	−0.4981**	−1.0587**	−0.0293*
	(0.0703)	(0.1587)	(0.0116)
SWB and wage variables			
Her life dissatisfaction ($LD_{f,t - 1}$)	0.1210**	0.2820**	0.0078*
	(0.0397)	(0.0907)	(0.0034)
His life dissatisfaction ($LD_{m, t - 1}$)	0.0555	0.1289	0.0029
	(0.0409)	(0.0970)	(0.0030)
She dissatisfied (binary variable)	−0.3654**	−0.8592**	−0.0145*
	(0.0933)	(0.2163)	(0.0065)
He dissatisfied (binary variable)	−0.2458*	−0.5592**	−0.0117*
	(0.0884)	(0.2082)	(0.0059)
Her wages	−0.0024	−0.0083	−0.0052
	(0.0602)	(0.1411)	(0.0041)
His wages	0.0620*	0.1367*	0.0017
	(0.0315)	(0.0688)	(0.0027)
She earns more	−0.1207	−0.2971	−0.0066
	(0.1408)	(0.3184)	(0.0104)
Interaction with She Dissatisfied	0.2941*	0.6371*	0.0094
	(0.1407)	(0.3194)	(0.0083)
Interaction with He Dissatisfied	−0.1297	−0.2533	0.0002
	(0.1310)	(0.2983)	(0.0089)
Wave fixed effects	Yes	Yes	Yes
Region fixed effects	Yes	Yes	Yes
Observations	10,149	10,149	10,149
Couples	1415	1415	1415

Random effects estimates. All variables are lagged one year. Constant included but not reported. Standard errors (in parentheses) are clustered on couples

*p < 0.05; **p < 0.01

Turning to the focal variables, we first discuss direction of effects and subsequently interpret their magnitude and significance when taken in conjunction with the interaction terms. The results of all three estimation techniques indicate that a dual-earner couple’s propensity to jointly migrate is reduced if either partner is less than fully satisfied with life. This finding reflects the inertia in decision-making when life is stressful or people are depressed. However, after accounting for this effect, the couple’s migration propensity is enhanced as her dissatisfaction with life increases; there is no such impact from his dissatisfaction with life.

There is no indication that the migration propensity changes according to who earns more in the household, although the probit and logit models suggest that higher male wages increase the migration propensity. The interaction term for she earns more with her being dissatisfied has the expected positive sign in each of the estimates. This coefficient is significant in the probit and logit equations, but not in the linear probability model; however, the non-linearities of the model mean that coefficient significance in the probit and logit models cannot by themselves be taken to indicate statistical significance once the base and interaction effects are considered together. To interpret the probit result further, we examine the marginal effect of the female partner being dissatisfied in cases where she either earns more or less.^5^

Table 6 presents the migration probabilities (and 95% confidence intervals) for the four possible cases from the probit estimates. The table shows the predicted probability for each combination of the interaction of “Female dissatisfied” and “She earns more” variables based on estimates from the probit regression reported in Table 5 while setting the remaining variables at their mean level. The first line of the table shows that when the female is satisfied (i.e. is at her maximal SWB), the couple’s estimated probability of moving is 4.5% if she is not the main breadwinner, but this probability falls to 3.0% if she is the dominant income earner. This result is as expected if earning more gives her greater bargaining power. The 1.5 percentage point reduction in migration propensity indicated by the probit estimates for this case compares with a 0.9 percentage point reduction indicated by the linear probability model. However, the confidence intervals in Table 6 overlap so the result cannot be treated as statistically significant (consistent with the insignificant interaction term in the linear probability model).

**Table 6:** Migration probabilities (%) for female dissatisfaction and she earns more

Female dissatisfied	She earns more	
	No (95% CI)	Yes (95% CI)
No	4.5% (3.3%, 5.6%)	3.0% (1.6%, 4.5%)
Yes	2.1% (1.7%, 2.5%)	2.6% (1.8%, 3.5%)

Results based on the probit estimates in Table 5 with other covariates set equal to their mean value

The second line of Table 6 indicates that when the woman is dissatisfied, the couple’s probability of migrating is 2.1% if she is not the main breadwinner, while the probability rises to 2.6% if she is the main income earner. Again the direction of this effect is as expected if earning more gives her greater bargaining power; however, the confidence intervals again overlap so this result can also not be treated as statistically significant. Thus while there is some indication that bargaining strength in relation to the migration choice may be present, we cannot reject the null hypothesis that the dominant income earner has no greater weight in the migration decision.

In further (unreported) estimates, we have replaced the binary ‘she earns more’, with a cardinal variable reflecting the female share of household wage earnings. When we do so, we again find no significant influence from this variable.

Table 5 indicates consistent results with respect to SWB both in terms of an inertia effect (from both partners) and an increasing migration propensity as her unhappiness in the current location increases. We further check the robustness of these results using different samples and different functional forms. First, we compare results from the same specification across different samples of couples according to their earnings.

The first four columns of Table 7 present results from the linear probability model for: all couples, dual-earner couples,^6^ male sole-earner couples, and female sole-earner couples. The table presents the results for the SWB and wage variables, but with all control variables included in each regression. Results for all couples are very similar to those for dual-earner couples and so are not discussed further. When the male is sole-earner, the importance of female life dissatisfaction remains an important determinant of migration, but the inertia effect of being dissatisfied is now attributed solely to the male, i.e. to the sole income earner. This suggests that, in this situation, he may be the dominant decision-maker (or prevaricator).

**Table 7:** Probability of joint migration for different couple earner types (GLS estimates)

	(1) All couples	(2) Dual earners	(3) Male sole earner	(4) Female sole earner	(5) Dual earners She earns more
SWB and wage variables					
Her life dissatisfaction ($LD_{f,t - 1}$)	0.0075**	0.0076*	0.0110*	0.0164	0.0098
	(0.0024)	(0.0033)	(0.0047)	(0.0116)	(0.0089)
His life dissatisfaction ($LD_{m,t - 1}$)	0.0016	0.0030	0.0003	0.0021	0.0016
	(0.0021)	(0.0029)	(0.0038)	(0.0051)	(0.0050)
She dissatisfied (binary variable)	−0.0150**	−0.0142**	−0.0140	−0.0084	−0.0034
	(0.0050)	(0.0062)	(0.0098)	(0.0154)	(0.0125)
He dissatisfied (binary variable)	−0.0154**	−0.0119*	−0.0177*	−0.0040	−0.0076
	(0.0046)	(0.0056)	(0.0089)	(0.0118)	(0.0106)
Her wages	−0.00052	−0.0048		−0.0040	0.0033
	(0.0031)	(0.0038)		(0.0081)	(0.0055)
His wages	−0.0002	0.0017	−0.0030		0.0102
	(0.0018)	(0.0026)	(0.0033)		(0.0111)
She earns more	−0.0085	−0.0080			
	(0.0088)	(0.0099)			
Interaction with She Dissatisfied	0.0079	0.0113			
	(0.0073)	(0.0079)			
Interaction with He Dissatisfied	0.0063	−0.0002			
	(0.0074)	(0.0084)			
Control variables	Yes	Yes	Yes	Yes	Yes
Wave fixed effects	Yes	Yes	Yes	Yes	Yes
Region fixed effects	Yes	Yes	Yes	Yes	Yes
Observations	15,084	10,693	3155	1552	2772
Couples	1660	1419	824	520	723

Random effects estimates. All variables are lagged one year. Constant included but not reported. Standard errors (in parentheses) are clustered on couples

*p < 0.05; **p < 0.01

When the female is sole-earner, her life dissatisfaction is no longer a significant determinant of migration. At face value, this result when combined with the previous result may suggest that a male sole-earner considers the female partner’s interest while a female sole-earner does not. Of note, however, is that the estimated coefficient on her life dissatisfaction rises substantially when she is sole-earner, but so too does the standard error (more than commensurately, possibly influenced by the reduced sample size). A similar, but less pronounced, pattern is seen when we subset on dual-earners where she earns more, shown in column (5). These patterns are consistent with a greater diversity of migration considerations in relation to her life dissatisfaction when she is the sole-earner or the dominant income earner compared with other cases.

As a robustness test of the role of labour force status, we added two terms to the linear probability model for dual-earners. These terms indicate whether the female or male partner respectively became redundant or was fired over the year in which migration occurred. Neither of these terms was a significant predictor of migration when added to the equation.^7^

In Table 8, we explore different functional forms for the life dissatisfaction variables. We do so since we have hitherto treated this variable as being cardinal whereas it is measured as an ordinal variable. When dealing with the ordinal values, we restrict the sample to include only observations for which life dissatisfaction is no greater than 5 given the miniscule number of observations (which are likely to be highly idiosyncratic) above that value. To keep the focus manageable, we confine our attention to dual-earner couples and drop the interaction terms (which, when included, are not statistically significant). Column (1) of Table 8 is based on the (cardinal) dual-earner equation from Table 7 with the slightly restricted sample (though the number of couples is unchanged). Results are consistent with the prior estimates for dual-earner couples.

**Table 8:** Probability of joint migration for dual earner couples: cardinal and ordinal specifications

	(1)	(2)	(3)
Her life dissatisfaction ($LD_{f,t - 1}$)	0.0067*		
	(0.0032)		
His life dissatisfaction ($LD_{m,t - 1}$)	0.0015		
	(0.0029)		
She dissatisfied (binary variable)	−0.0102	−0.0025	−0.0017
	(0.0057)	(0.0040)	(0.0039)
He dissatisfied (binary variable)	−0.0102	−0.0082*	−0.0084*
	(0.0053)	(0.0040)	(0.0039)
Her life dissatisfaction: $LD_{f,t - 1}$ = 2		0.0021	
		(0.0038)	
Her life dissatisfaction: $LD_{f,t - 1}$ = 3		0.0057	
		(0.0082)	
Her life dissatisfaction: $LD_{f,t - 1}$ = 4		0.0391*	
		(0.0197)	
Her life dissatisfaction: $LD_{f,t - 1}$ = 5		0.0406	
		(0.0302)	
His life dissatisfaction: $LD_{m,t - 1}$ = 2		0.0009	
		(0.0044)	
His life dissatisfaction: $LD_{m,t - 1}$ = 3		−0.0094	
		(0.0064)	
His life dissatisfaction: $LD_{m,t - 1}$ = 4		0.0359	
		(0.0197)	
His life dissatisfaction: $LD_{m,t - 1}$ = 5		−0.0084	
		(0.0226)	
Her life dissatisfaction: $LD_{f,t - 1}$ = 4 to 5			0.0371*
			(0.0170)
His life dissatisfaction: $LD_{m,t - 1}$ = 4 to 5			0.0250
			(0.0156)
Her wages	−0.0049	−0.0047	−0.0047
	(0.0038)	(0.0038)	(0.0038)
His wages	0.0016	0.0016	0.0016
	(0.0025)	(0.0023)	(0.0025)
She earns more	−0.0004	−0.0003	−0.0004
	(0.0051)	(0.0051)	(0.0051)
Control variables	Yes	Yes	Yes
Wave fixed effects	Yes	Yes	Yes
Region fixed effects	Yes	Yes	Yes
Observations	10,646	10,646	10,646
Couples	1419	1419	1419

GLS random effects estimates. All variables are lagged one year. Constant included but not reported. Standard errors (in parentheses) are clustered on couples

*p  < 0.05; **p < 0.01

Column (2) of the table retains the dissatisfied binary variables while replacing the cardinal life dissatisfaction variables with their ordinal counterparts (in which life dissatisfaction =1 is the base category). For females, the estimated coefficients show a rising effect of dissatisfaction on migration propensity as the degree of dissatisfaction increases (with $LD_{i,t - 1}$= 4 being statistically significant). For males, no clear pattern emerges.

Given the patterns in column (2) and in Table 4, we group together moderately dissatisfied people (1 ≤ $LD_{i,t - 1}$≤ 3) and strongly dissatisfied people (4 ≤ $LD_{i,t - 1}$≤ 5) into two groups, with the former being the base category. Results using these groupings are shown in column (3). Females who register strong dissatisfaction with their lives increase the probability of migration by 3.7 percentage points. As in prior results, the magnitude of this effect is stronger than for the corresponding value for males (2.5 percentage points) and the latter is not statistically significant. The results using the ordinal life dissatisfaction data are therefore consistent with those using the cardinal representation.

## Discussion and conclusions

Preston and Grimes (2019), investigating the *ex post* effects of migration, found that when couples migrate, men’s wages tend to rise (while women’s wages do not) but it is women who on average gain greater SWB (while men do not). Using the same data, our *ex ante* results, predicting whether a dual-earner couple migrates, show some similar patterns. Women’s SWB (relative to their maximal level) helps predict migration (while men’s SWB does not), and probit and logit estimates indicate that men’s wages in their current location helps predict migration (while women’s do not). Thus the *ex post* results reported in prior work correspond to the core *ex ante* predictors for dual-earner couples that we estimate here.

However, when we examine these core results further, several more nuanced findings emerge. First, the positive impact of female life dissatisfaction is most pronounced at high levels of dissatisfaction ($LD_{f,t - 1}$ = 4 to 5). Second, people who are most satisfied with life ($LD_{i,t - 1}$ = 0) have a high propensity to migrate relative to people who are dissatisfied, consistent with inertia in decision-making when people suffer from conditions such as stress or depression. Third, we find some indicative (but not statistically significant) evidence that the results for the female partner’s SWB are magnified in dual-earner couples where she earns more than her partner, consistent with increased bargaining power for the female partner in this case. Fourth, when the male is the sole earner in a couple, the impact of female life dissatisfaction is greater than is the case for dual-earner couples implying that even though he is the labour market participant, the migration decision may still be influenced by her life dissatisfaction. Fifth, when the female is the sole or dominant earner in the couple, the estimated mean effect of her life dissatisfaction is even greater but the increased standard error—reflecting either the smaller sample size or greater heterogeneity of response—leaves this effect statistically insignificant.

Overall, the evidence presented here is complementary to standard labour market explanations of couple migration which emphasise the couple’s (and especially the male’s) wage and employment prospects that can arise from migration. While we find some limited evidence that male (pre-migration) wages may influence the migration choice, our results with respect to the impact of SWB highlight that prospective labour market factors are not the sole (or necessarily the dominant) determinant of whether a couple chooses to leave their existing location; the female’s satisfaction—or dissatisfaction—with life also plays an important role in the couple’s migration choice.

Our analysis is restricted to different-sex couples, so we cannot generalise these results to migration decisions of same-sex couples. Furthermore, our dataset reveals whether an individual or couple has migrated within a year, but not whether that individual or couple considered the possibility of migration but ultimately decided not to. We are unaware of any national panel datasets which allow for this level of detail, but if the information were available it would add further richness to the nature of couples’ decision-making with respect to migration.

Our dataset is from Australia, a developed country that is moderately egalitarian across the sexes. For instance, the OECD calculates that on average over our sample period, the gender wage gap in Australia was identical to the OECD average (at 15%).^8^ The UNDP rates Australia as 8^th^ out of 66 ‘very high human development’ countries according to its Gender Development Index constructed as the ratio of the Human Development Index calculated separately for females and males.^9^ An extension of our work would be to test whether our findings differ according to different cultural norms, values and levels of development. Even within Australia, for instance, Grosjean and Khattar (2019) show (using HILDA responses paired with historical spatial sex-ratio data) that cultural norms with respect to household allocation of responsibilities differ regionally. It would be instructive to understand how the nature of decision-making within couples with respect to migration—including the role in the decision of each couple member’s SWB—differs both across cultures and across states of development.

## Footnotes

1 The present COVID-19 pandemic has severely restricted travel and migration, but we expect this to be temporary.

2 Economists have focused primarily on evaluative wellbeing rather than other measures of wellbeing (i.e. those based on affect or eudaimonia), as conceptually it is closer to a measure of utility relevant to economic decisions (Dolan et al., 2011).

3 For studies of couples’ residential location choice within an urban area, see Mok (2007) and Picard et al. (2013).

4 Although the bargaining model could equally apply to same-sex couples, there were only a handful of observations for these couples. We therefore exclude them from our analysis.

5 This calculation incorporates all the information from the “dissatisfied” and “she earns more” variables, including their interactions, and is calculated using the margins command within Stata. The marginal effects for logit estimates display a similar pattern.

6 The sample size for these estimates is slightly larger than in Table 5 where the sample was restricted to be the same across all three estimation techniques.

7 As a further robustness check, we tested whether sub-setting on samples in which the female partner is in the labour force in both *t-1* and *t* affected the results, finding no substantive differences from the results presented in Table 7.

8 Source: http://data.oecd.org/earnwage/gender-wage-gap.htm

9 Source: http://hdr.undp.org/en/content/gender-development-index-gdi
